# Allo-HCT with post-transplant cyclophosphamide in older adults: similar safety and a viable option compared to younger adults

**DOI:** 10.3389/fimmu.2025.1678899

**Published:** 2026-01-07

**Authors:** Gorka Pinedo, María Suárez-Lledó, Maite Antonio, Laia Guardia, Berta Solé, Paola Charry, Joan Cid, Miquel Lozano, Alejandra Pedraza, Jordi Esteve, Enric Carreras, Francesc Fernández-Avilés, Carmen Martínez, Montserrat Rovira, María Queralt Salas

**Affiliations:** 1 Hematology Department, Cruces University Hospital, Bilbao, Spain; 2 Hematopoietic Transplantation Unit, Hematology Department, Institute of Cancer and Blood Diseases Institut del Càncer i Malalties de la Sang (ICAMS), Hospital Clínic de Barcelona, Barcelona, Spain; 3 Institut d’Investigacions Biomèdiques August Pi i Sunyer (IDIBAPS), Barcelona, Spain; 4 Universitat de Barcelona, Barcelona, Spain; 5 Geriatric-Oncology Unit, Institute of Cancer and Blood Diseases Institut del Càncer i Malalties de la Sang (ICAMS), Barcelona, Spain; 6 Apheresis and Cellular Therapy Unit, Hemotherapy and Hemostasis Department, Institute of Cancer and Blood Diseases Institut del Càncer i Malalties de la Sang (ICAMS), Hospital Clínic de Barcelona, Barcelona, Spain; 7 Fundació i Institut de Reserca Josep Carreras Contra la Leucèmia, Barcelona, Spain

**Keywords:** older patients, age-related complications, allo-HCT, PTCY-based, outcomes

## Abstract

**Introduction:**

Given the increasing number of older patients undergoing allo-HCT with post-transplant cyclophosphamide (PTCy)-based prophylaxis, a dedicated evaluation of its safety in this population is warranted.

**Methods:**

We retrospectively analyzed 353 consecutive patients who underwent first allo-HCT with PTCy between 2014 and 2024. Patients were stratified into three age groups: ≤40 (24.4%), 41–64 (51.8%), and ≥65 (23.8%).

**Results:**

Median age was 53 years (range 18–75). Older patients mostly received RIC regimens and matched unrelated donors and younger MAC and haplo-HCT. Neutrophil and platelet engraftment occurred at amedian of 18 and 17 days, without differences among age groups. The incidence of grade II-IV aGVHD at day +100 was 22.4% with no differences according to age ranges (Day +100: 16.3%, 24.0% and 25.0%, P = 0.246) and moderate-severe cGVHD in 7.4%, with incidence significantly lower in patients ≥65 years (2-year: 3.1% vs. 12.1% and 4.8%; P = 0.023). At 2-years, OS rates were 79.5% for ≤40, 73.9% for 41–64, and 57.9% for ≥65 years (P = 0.001). NRM rates were 7.1%, 15.6%, and 16.0% (P = 0.128), and relapse incidence rates were, respectively, 26.2%, 24.6% and 40.7% (P = 0.039).

**Discussion:**

Despite higher relapse rates leading to lower OS in older adults, similar NRM and comparable early toxicities support the feasibility of allo-HCT with PTCy in patients ≥65 years.

## Introduction

Allogeneic hematopoietic cell transplantation (allo-HCT) remains a potentially curative treatment for patients with high-risk hematologic malignancies (1). However, its success remains mitigated by significant risks of morbidity, mortality, and detrimental effects on quality of life. Over the past two decades, several advances in donor selection, transplant platforms and optimized supportive care have collectively decreased transplant-related toxicity (2–4). This evolution in allo-HCT practice has permitted the expansion of allo-HCT to older adults with notable clinical success, and these advances are northwardly considering that life expectancy has increased, and the number of older adults diagnosed with high-risk hematological disorders is an increasing demographic group.

The introduction of post-transplant cyclophosphamide (PTCy) for GVHD prophylaxis—originally developed for haploidentical transplants—has revolutionized allo-HCT by reducing GVHD incidence and severity while preserving the graft-versus-leukemia effect (5–8). Nevertheless, PTCy use has been also associated with non-desirable risks, including delayed engraftment, infections, delayed immune reconstitution, and potential cardiotoxicity.

Given the increasing number of older patients undergoing allo-HCT and the growing use of PTCy-based prophylaxis, dedicated evaluations of its safety and efficacy in this population are still limited (9, 10). Since 2013, starting in the haploidentical HCT setting, PTCy-based prophylaxis has been progressively integrated into our practice and is now the standard approach at our institution regardless of donor type. Building on these data, the present study retrospectively analyzes the outcomes of all consecutive adult patients who underwent a first allo-HCT with PTCy-based prophylaxis at our institution between January 2014 and June 2024. The study also explores whether transplant outcomes and toxicity differ according to chronological age.

## Methods

### Patient selection

The study includes the 353 consecutive adults who underwent their first allo-HCT with PTCY-based prophylaxis at our Institution between January 2014 and June 2024. All patients received peripheral blood stem cell (PBSC) grafts. Data were collected retrospectively and updated in January 2025. The present investigation was approved by the Ethics Committee of the HCB and conducted following standards set forth by the Declaration of Helsinki.

### Eligible criteria for allo-HCT and donor selection

General eligibility criteria for allo-HCT and donor selection algorithm were homogeneous during the study period and did not vary according to chronological age. All patients aged above 17 years with a Karnofsky performance score (KPS) ≥60% and without uncontrolled comorbidities were considered potentially eligible for allo-HCT. The general criteria for pre-transplant organ function are described in **Supplementary Material**.

High resolution DNA typing for HLA-A, -B, -C, -DRB1, and -DQB1 was conducted in recipients and donors. An HLA-matched sibling donor (MSD) followed by a 10/10 HLA-matched unrelated donor (MUD) were preferred upfront, and alternative stem cell sources as 9/10 MMUD or haploidentical donors are considered the third and fourth choices. Donor age and other variables were taken into consideration but always after HLA compatibility.

### Main allo-HCT information and GVHD prophylaxis

Reduced-intensity conditioning (RIC) regimens were primarily chosen for patients older than 55 years, those with prior HCT, or significant comorbidities. Myeloablative conditioning (MAC) regimens included fludarabine (30 mg/m²/day for 4 days) combined with high-dose busulfan (3.2 mg/kg/day IV for 4 days) or 12 Gy total body irradiation (TBI). RIC regimens used lower doses of busulfan (3.2 mg/kg/day IV for 3 days) or 8 Gy TBI with fludarabine. All haploidentical HCT recipients also received 2 Gy TBI when TBI was not part of conditioning. Sequential regimens were used selectively for myeloid malignancies.

Based on this comment, the following information has been clarified:

All patients included received PTCY-based GVHD prophylaxis. From 2013 to early 2017, this regimen consisted of PTCY 50 mg/kg on days +3 and +4, MMF (15 mg/kg every 8 h, from day +5 to +30), and tacrolimus (0.3 mg/kg/day IV, starting on day +5, continued to day +90). Between 2017 and 2022, MMF was progressively discontinued in HLA-matched and mismatched unrelated donor (MMUD) transplants, and patients received only PTCY plus tacrolimus. In patients with pre-transplant renal dysfunction, PTCY was combined with sirolimus (6 mg loading, then 2 mg/day) and MMF instead of tacrolimus. Tacrolimus or sirolimus were adjusted to maintain therapeutic levels and continued until day +90, then tapered and discontinued by day +180 in the absence of GVHD.

Unmanipulated T-cell replete PBSC grafts were infused on day 0 with a maximum CD34+ cell dose capped at 8 × 10^6^/Kg and a minimum cell dose of 2.5 x10^6^/Kg. Graft cryopreservation was allowed depending on donor availability and consistent during COVID19 pandemic. Since November 2021, granulocyte colony stimulating factor (G-CSF) is administered subcutaneously at a dose of 300 mcg daily from day +7 until absolute neutrophil count exceeds 1.5 x 10^9/L in all cases.

### Supportive care and key definitions

Antimicrobial prophylaxis included levofloxacin 500 mg daily from day +1 until neutrophil engraftment, fluconazole 400 mg daily from day +1 until day +60, acyclovir 800 mg twice daily from day +1 until 1 year after allo-HCT, and either trimethoprim-sulfamethoxazole 160/800 mg three times per week, or inhaled pentamidine 300 mg monthly until the achievement of peripheral blood CD4+ cell count> 200 cells/ml. Since November 2021, CMV prophylaxis with 480 mg of letermovir has been implemented for all CMV-seropositive patients undergoing allo-HCT until day +100.

Acute GVHD (aGVHD) was graded according to the MAGIC (Mount Sinai Acute GvHD International Consortium) criteria, while chronic GVHD (cGVHD) was graded based on the 2014 NIH Consensus Criteria (11, 12). Patients with GVHD were managed uniformly throughout the study period. Additional definitions can be found in the **Supplementary Material**.

### Statistical analysis

For the study conduction, patients were classified into three groups according to the main explanatory variable: chronological age (younger ≤40 years, middle-aged 41–64 years, and older ≥65 years). The primary outcome variables were overall survival (OS), non-relapse mortality (NRM), cumulative incidence of relapse (CIR), and GVHD-Free/RFS (GRFS).

Descriptive statistics were used to summarize categorical and continuous variables with counts, percentages, medians, and ranges. Time to event analysis was performed from the date of transplant to the date of event or last follow-up. OS and GRFS were calculated using the Kaplan-Meier method. NRM and CIR were estimated using the competing risk analysis by Fine and Gray, considering relapse as a competing event for NRM and death without relapse as competing event for CIR. The cumulative incidence of GVHD was calculated while accounting for death and relapse as competing events, and the rest of post-transplant events were estimated considering death as a competing event. The impact of the main explanatory variable (age groups) on outcomes was explored using univariate and multivariate regression analyses (UVA and MVA). Multivariable analyses were performed using both Cox proportional hazards and Fine-Gray competing risks models, including all variables with a p-value <0.1 in univariate analysis, as well as variables considered clinically relevant for allo-HCT outcomes. No formal multiple comparison adjustment was applied, since all covariates were analyzed simultaneously within a single multivariable model. All p-values were 2-sided, and for statistical analyses a p-value of <0.05 was considered to indicate statistical significance. Statistical analysis was performed using EZR (13).

## Results

### Baseline information

The study included 353 patients who were classified into the following three age groups: younger ≤40 years (n=86, 24.4%), middle-aged 41–64 years (n=183, 51.8%), and older ≥65 years (n=84, 23.8%).

As shown in **Table 1**, the median age of the study cohort was 53 years (range 18–75), and 60.1% of patients were male. The most common baseline diagnoses were acute myeloid leukemia (AML) (n=127, 36.0%), myelodysplastic syndromes (MDS)/chronic myelomonocytic leukemia (CMML) (n=66, 18.7%), and acute lymphoblastic leukemia (ALL) (n=67, 19.0%).

**Table 1:** Baseline characteristics of the study cohort and of patients.

	Overall N=353	≤40 years N=86 (24.4)	41–64 years N = 183 (51.8)	≥65 years N= 84 (23.8)	P value
Median age, years (Range)	53 (18-75)	27 (18-40)	53 (40-65)	67 (66-75)	<0.001
Sex					
Male	212 (60.1)	48 (55.8)	111 (60.7)	53 (63.1)	0.608
Female	131 (39.9)	38 (44.2)	72 (39.3)	31 (36.9)	
Baseline characteristics					
History of smoking	101 (252)	12 (14)	64 (36.6)	31 (36,9)	0.001
HTA	79 (22.4)	2 (2.3)	42 (23)	37 (44)	<0.001
Mellitus diabetes	31 (8.8)	1 (1.2)	18 (9.8)	12 (14.3)	0.001
DLP	46 (13.0)	2 (2.3)	24 (13.1)	22 (26.2)	<0.001
Cardiopathy	36 (10.2)	5 (5.8)	13 (7.1)	18 (21.4)	0.001
Baseline diagnosis					
AML	127 (36.0)	29 (33.7)	60 (32.8)	38 (45.2)	0.001
MDS/CMML	66 (18.7)	1 (1.2)	37 (20.2)	28 (33.3)	
MPN	26 (7.4)	6 (6.9)	11 (6.0)	9 (10.7)	
ALL	67 (19.0)	29 (33.7)	34 (18.6)	4 (4.8)	
Lymphoproliferative disorders	44 (12.5)	18 (20.9)	24 (13.1)	2 (2.4)	
Plasma Cell Discrasias	13 (3.7)	1 (1.2)	11 (6.0)	1 (1.2)	
Other	10 (2.8)	2 (2.3)	6 (3.3)	2 (2.4)	
- Malignant	8 (2.3)	1 (1.2)	5 (2.7)	2 (2.4)	
- Non-Malignant	2 (0.5)	1 (1.2)	1 (0.5)	0 (0.0)	
Disease risk index					
Low/Intermediate	251 (71.1)	61 (70.1)	136 (74.3)	54 (64.3)	0.089
High/Very High	88 (24.9)	23 (27.7)	37 (20.2)	28 (33.3)	
Not applicable	14 (4.0)	2 (2.3)	10 (5.5)	2 (2.4)	
HCT-CI					
>3	80 (22.7)	12 (14.0)	43 (23.5)	25 (29.8)	0.045
Karnofsky performance status					
<90%	82 (23.2)	18 (20.9)	45 (24.6)	19 (22.6)	0.794
Conditioning intensity					
MAC	149 (43.1)	68 (79.1)	81 (44.3)	0 (0.0)	<0.001
RIC	204 (56.9)	18 (20.9)	102 (55.7)	84 (100.0)	
Conditioning intensity (extended)					
Flu/Bu4 (+/- 2 Gys TGI)	77 (21.8)	31 (36.0)	46 (25.1)	0	<0.001
Flu/TGI12	59 (16.7)	32 (37.2)	27 (14.6)	0	
Flu/Bu3 (+/- 2 Gys TGI)	122 (34.6)	5 (5.8)	62 (33.9)	55 (65.5)	
Flu/TGI8	17 (4.8)	1 (1.2)	12 (6.6)	4 (4.8)	
Flu/Treo10 (+/- 2 Gys TGI)	16 (4.5)	2 (2.3)	2 (1.1)	12 (14.3)	
Others	64 (18.1)	17 (19.8)	34 (18.6)	13 (15.5)	
GVHD prophylaxis					
PTCY-TK-MMF	83 (23.5)	33 (39.8)	32 (38.6)	18 (21.7)	–
PTCY-TK	259 (73.4)	53 (20.5)	143 (55.2)	63 (24.3)	
PTCY-Sir-MMF	9 (2.5)	1 (11.1)	6 (66.7)	2 (22.2)	
Donor/product type					
10/10 MRD	54 (15.2)	10 (11.6)	34 (18.6)	10 (11.9)	0.001
10/10 MUD	124 (35.1)	26 (30.2)	64 (35.0)	34 (40.5)	
9/10 MUD	103 (29.2)	19 (22.1)	61 (33.3)	23 (27.4)	
Haploidentical	72 (20.4)	31 (36.0)	24 (13.1)	17 (20.2)	
Stem cell product					
Median CD34+ cell dose (IQR)	6.0 (5 - 7.2)	6.4 (5.3 - 7.4)	5.9 (4.8 - 7.1)	6.2 (4.5 - 7.1)	0.268
Systematic use of G-CSF*					
Yes	98 (27.8)	23(26.7)	39 (21.3)	36 (42.9)	0.026
Systematic use of letermovir**					
Yes	98 (27.8)	24 (27.8)	38 (20.8)	36 (42.9)	0.01
Median Follow-up (months) (IQR)	27.4 (10.0-55.1)	31.6 (14.4-48.8)	33.5 (13.2-58.8)	16.7 (7.5-34.7)	0.012

*From Day +7 to neutrophil engraftment. All patients undergoing allo-HCT after November 2021.

**From Day +7 to day +100. Only in CMV-seroposive patients. Prophylaxis started on November 2021.

AML: acute myeloid leukemia; MDS: myelodysplastic syndrome; MPN: myeloproliferative neoplasms; ALL: acute lymphoblastic leukemia; MSD: matched sibling donor; MUD: matched unrelated donor.

The distribution of baseline diagnosis varied significantly across age groups (P = 0.001). While AML and MDS were more prevalent in older patients, ALL was the leading indication for allo-HCT in younger individuals. As expected, comorbidities, including cardiovascular risk factors or prior cardiac toxicity, were more prevalent in older adults than in younger and middle-aged ones (HCT-CI >3: 14.0% vs. 23.5% and 29.8%, P = 0.045). However, KPS <90% was observed in 23.2% of cases, with no differences among age groups (20.9%, 24.6%, and 22.6%, P = 0.794).

The median follow-up in the study cohort was 28 months (IQR 10–55). Follow-up times differed between age groups, reflecting both the increasing proportion of older adults undergoing allo-HCT in recent years (**Supplementary Figure 1**) and their shorter life expectancy compared to younger patients.

### Allo-HCT characteristics

Allo-HCT information is also described in **Table 1**. Older adults received RIC regimens while younger patients predominantly received MAC regimens (P<0.001). Donor type differed between groups: older adults received frequent grafts from 10/10 MUDs (n=34, 41%) while younger patients tended to receive grafts from haploidentical donors (n=31, 36%).

Three-hundred forty-two (97%) patients received PTCY with Tacrolimus (and with MMF if haploidentical donors), while 9 (2.5%) patients with pre-transplant kidney dysfunction received a PTCY combined with sirolimus and MMF (P<0.001). PBSC were infused in all cases, with comparable median CD34+ cell doses across groups (6.4, 5.9, and 6.2 x 10^9^ cells respectively, P = 0.268).

### Engraftment information and allo-HCT results

Main post-transplant information is shown in **Table 2**. The duration of the HCT hospitalization was 29 days (IQR: 23-38). Overall, neutrophil and platelet engraftment occurred at a median of 18 days (IQR: 7 - 22), and 17 days (IQR: 13 - 27), respectively, with no differences among age groups (P = 0.787 and P = 0.157, respectively). Graft failure (GF) occurred in 14 (4.0%) patients, with no variation across age groups (P = 0.263). Among them, eight (57.1%) patients underwent a second allo-HCT and two (14.3%) received a CD34+ cell dose boost and successfully engrafted. However, seven (70.0%) of these ten patients consecutively died due to post-transplant complications.

**Table 2:** Main post-transplant information.

	Overall	≤40 years	41–64 years	≥65 years	
	N=353	N=86 (24.4)	N = 183 (51.8)	N= 84 (23.8)	P value
Median days to Admission (IQR)*	29 (23-38)	27 (22-36)	30 (24-41)	30 (23-37)	0.3
Median days to Engraftment (IQR)					
Neutrophil	18 (15-22)	18 (15-21)	18 (16-22)	18 (16-22)	0.787
Platelets	17 (13-27)	16 (12-26)	18 (13-27)	19 (15-28)	0.157
Graft failure	14 (4)	1 (1.2)	10 (5.5)	3 (3.6)	0.263
Primary	7 (2)	0 (0)	4 (2.2)	3 (3.6)	0.118
Non-infectious complications					
VOD	7 (2)	2 (2.3)	3 (1.6)	2 (2.4)	0.89
TMA	15 (4.2)	4 (4.7)	10 (5.5)	1 (1.2)	0.268
Main post-transplant complications, % (95% CI)					
Cumulative Incidence of GVHD, % (95% CI)					
Day +100 Grade 2–4 aGVHD	22.4 (18.2-26.9)	16.3 (9.4-24.9)	24.0 (18.1-30.5)	25.0 (16.3-34.7)	0.246
Day +100 Grade 3–4 aGVHD	10.8 (7.8-14.3)	7.0 (2.8-13.7)	10.4 (6.5-15.3)	15.5 (8.7-24.1)	0.145
2-year Mod/Sev cGVHD	10.8 (7.8-14.3)	4.8 (1.6-11.0)	12.1 (7.5-17.7)	3.1 (0.6-9.7)	0.023
Cumulative Incidence of Infectious Complications, % (95% CI)					
Day +30 BSI	39.4 (34.3-44.4)	29.1 (19.9-38.9)	43.2 (35.9-50.2)	41.7 (31.0-52.0)	0.104
Day +180 CMV reactivation (all)	37.7 (32.6-42.7)	34.9 (25.0-45.0)	43.7 (36.4-50.8)	27.4 (18.3-37.2)	0.024
Day +180 CMV reactivation (with letermovir prof, n=99)	8.1 (3.8-14.5)	4.2 (0.3-18.0)	15.4 (6.1-28.5)	2.8 (0.2-12.6)	0.091
Day +180 CMV disease (all)	5.9 (3.8-8.8)	8.1 (3.6-15.2)	6.0 (3.2-10.1)	3.6 (0.9-9.2)	0.406
Day +180 CMV disease (with letermovir prof,n=99)	2.0 (0.4-6.5)	4.2 (0.3-18.0)	2.6 (0.2-11.7)	0	0.508
Day +180 Grade 2–4 BK HC	12.7 (9.5-16.5)	14.0 (7.6-22.2)	13.7 (9.1-19.1)	9.5 (4.4-17.0)	0.599
Day +180 VHH6 React/Inf	16.7 (13.0-20.8)	20.9 (13.0-30.1)	16.4 (11.4-22.1)	13.1 (6.9-21.3)	0.299
Day +180 Fungal infection	7.6 (5.2-10.7)	5.8 (2.1-12.2)	8.7 (5.2-13.4)	7.1 (2.9-14.0)	0.39
Cumulative Incidence of Complications, % (95% CI)					
Day +180 ICU Admission	13.3 (10.0-17.1)	15.1 (8.5-23.5)	13.1 (08.7-18.5)	11.9 (6.1-19.9)	0.902
1-Year Cardiac Events	10.5 (7.6-14.0)	5.8 (2.1-12.2)	10.4 (6.5-15.3)	15.5 (8.7-24.1)	0.123
Main outcomes, % (95% CI)					
Outcomes					
Relapse	106 (30)	22 (25.6)	51 (27.9)	33 (39.3)	0.098
Dead	119 (33.7)	21 (24.4)	59 (32.2)	39 (46.4)	0.008
Causes of death					
Disease relapse	59 (16.7)	13 (15.1)	27 (14.7)	19 (22.6)	0.656
Infection	27 (7.6)	2 (2.3)	17 (9.2)	8 (9.5)	
GVHD	9 (2.5)	1 (1.1)	5 (2.7)	3 (3.5)	
Graft failure	5 (1.4)	1 (1.1)	2 (1)	2 (2.3)	
Secondary Malignancy	3 (0.8)	0 (0)	2 (1)	1 (1.1)	
VOD/MAT	2 (0.5)	1 (1.1)	0 (0)	1 (1.1)	
Other (toxicity, cardiopathy, hemorrhage)	5 (1.4)	2 (2.3)	1 (0.5)	2 (2.3)	
Overall Survival					
1-year	77.6 (72.8-81.6)	83.3 (73.5-89.8) 79.5 (69.0-86.7)	79.6 (73.0-84.8) 73.9 (66.6-79.8)	67.2 (55.9-76.2)	0.001
2-year	71.5 (66.4-76.1)	7.1 (2.9-13.9)	14.3 (9.6-19.8)	57.9 (45.9-68.1)	0.001
Non-Relapse Mortality					
1-year	12.6 (9.3-16.3)	7.1 (2.9-13.9)	15.6 (10.7-21.3)	14.5 (7.9-23.0)	0.128
2-year	13.6 (10.2-17.4)	73.1 (62.4-81.2) 66.7 (55.5-75.7)	69.2 (61.9-75.4)	16.0 (8.9-24.8)	0.128
Relapse-Free Survival					
1-year	65.8 (60.5-70.5)	19.8 (12.1-28.9)	59.8 (52.1-66.7)	50.8 (39.5-60.9)	0.002
2-year	57.6 (52.1-62.7)	26.2 (17.3-36.0)	16.5 (11.5-22.3)	43.3 (32.2-54.0)	0.002
Cumulative Incidence of Relapse					
1-year	21.7 (17.5-26.1)	62.6 (51.4-71.9)	24.6 (18.5-31.3)	34.8 (24.7-45.1)	0.038
2-year	28.8 (24.1-33.7)	57.4 (46.1-67.2)	58.2 (50.7-65.0)	40.7 (29.8-51.3)	0.038
GRFS					
1-year	56.4 (51.0-61.4)		46.3 (38.7-53.5)	46.1 (35.1-56.3)	0.012
2-year	46.9 (41.5-52.2)			37.4 (26.8-48.0)	0.012

*Calculated from day 0 to discharge date.

**Figure 1:**
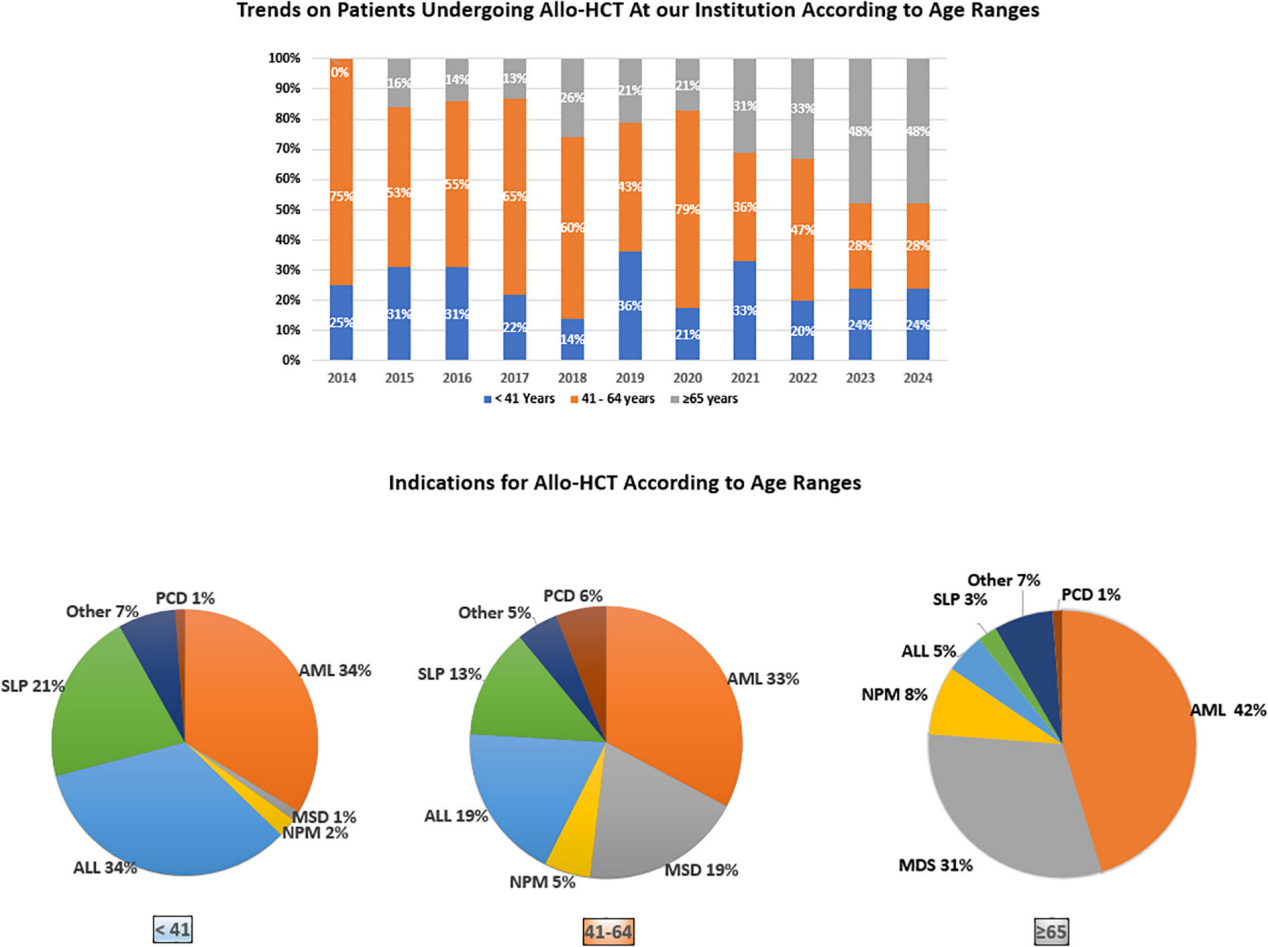
Dynamics of allo-HCT performed in patients across age ranges. Annual trends and main indications for allo-HCT stratified by age groups (younger ≤40 years, middle-aged 41–64 years, older ≥65 years).

The incidence of veno-occlusive disease (2%) and thrombotic microangiopathy (4.2%) was low, and did not differ significantly between groups (P = 0.890 and P = 0.268, respectively). Overall, the cumulative incidence of ICU admission at day +180 was 13.3% (95% CI 10.0% - 17.1%), while the cumulative incidence of cardiac events within 1-year post-transplant was 10.5% (95% CI 7.6% - 14.0%). Neither of these complications showed significant differences across age groups (P = 0.902 and P = 0.123, respectively) (**Figure 1**).

### Graft versus-host disease

**Figure 2:**
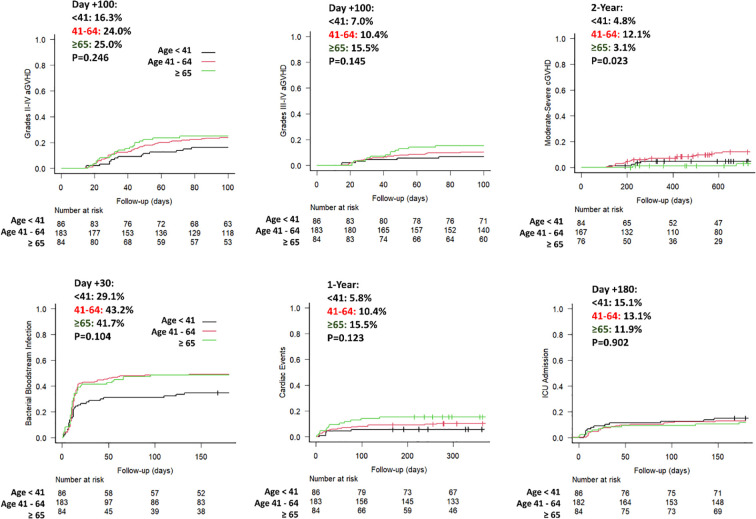
GVHD incidences and main complications. Cumulative incidence of acute and chronic GVHD, and distribution of main post-transplant complications across age groups (younger ≤40 years, middle-aged 41–64 years, older ≥65 years).

Acute GVHD was diagnosed in 170 patients, at a median of 39 days after allo-HCT. The cumulative incidences of grade II-IV and grade III-IV aGVHD at day +100 were 22.4% (95% CI 18.2%–26.9%) and 10.8% (95% CI 7.8%–14.3%), respectively, without significant differences between age groups (P = 0.246 and P = 0.145, respectively). Chronic GVHD was diagnosed in 89 (25.2%) patients, with moderate-to-severe forms in 26 (7.4%) patients, at a median of 230 days (IQR: 175–493) after stem cell infusion. Notably, older patients had a lower 2-year cumulative incidence of moderate-to-severe cGVHD (3.1%) compared with middle-aged patients (12.1%), while the difference with younger patients (4.8%) was not statistically significant (P = 0.023 for overall comparison; pairwise comparisons: older vs younger, P = 0.089; older vs middle-aged, P = 0.041) (**Figure 2**).

There were no differences in clinical manifestations, and treatment approaches were not tailored according to age. All patients were managed with curative intent. However, seven patients died directly due to active aGVHD.

### Infectious complications during the early post-transplant period

Infectious complications were prevalent in all groups irrespective of the patient’s age. As reported in **Table 2**, a higher incidence of bacterial bloodstream infections (BSI) was observed in older and middle-aged patients than in younger ones (Day +30 cumulative incidence: 41.7% and 43.2% vs. 29.1%, respectively, P = 0.104). The cumulative incidence of CMV reactivation was lower in the older patients than in younger and middle-aged ones (Day +180: 27.4% vs. 43.7% and 34.9%, P = 0.024). Lastly, by day 180, the global cumulative incidences of grade 2–4 BK hemorrhagic cystitis, VHH6 reactivation or infection, and probable or proven invasive pulmonary fungal infections were 12.7% (95% CI 9.5–16.5), 16.7% (95% CI 13.0–20.8), and 7.6% (95% CI 5.2–7.5), respectively, with no differences between age groups (P = 0.599, P = 0.299, and P = 0.390, respectively).

### Main outcomes

During the study period, 106 (30.0%) patients manifested disease relapse and 119 (33.7%) died, primarily due to relapse and infection. Causes of death are reported in **Table 2**. Overall, the 3-year OS, NRM and CIR rates were 65.4% (95% CI 59.8%–70.5%), 14.8% (95% CI 11.2%–18.8%) and 30.8% (95% CI 25.8%–35.8%), respectively.

**Figure 3:**
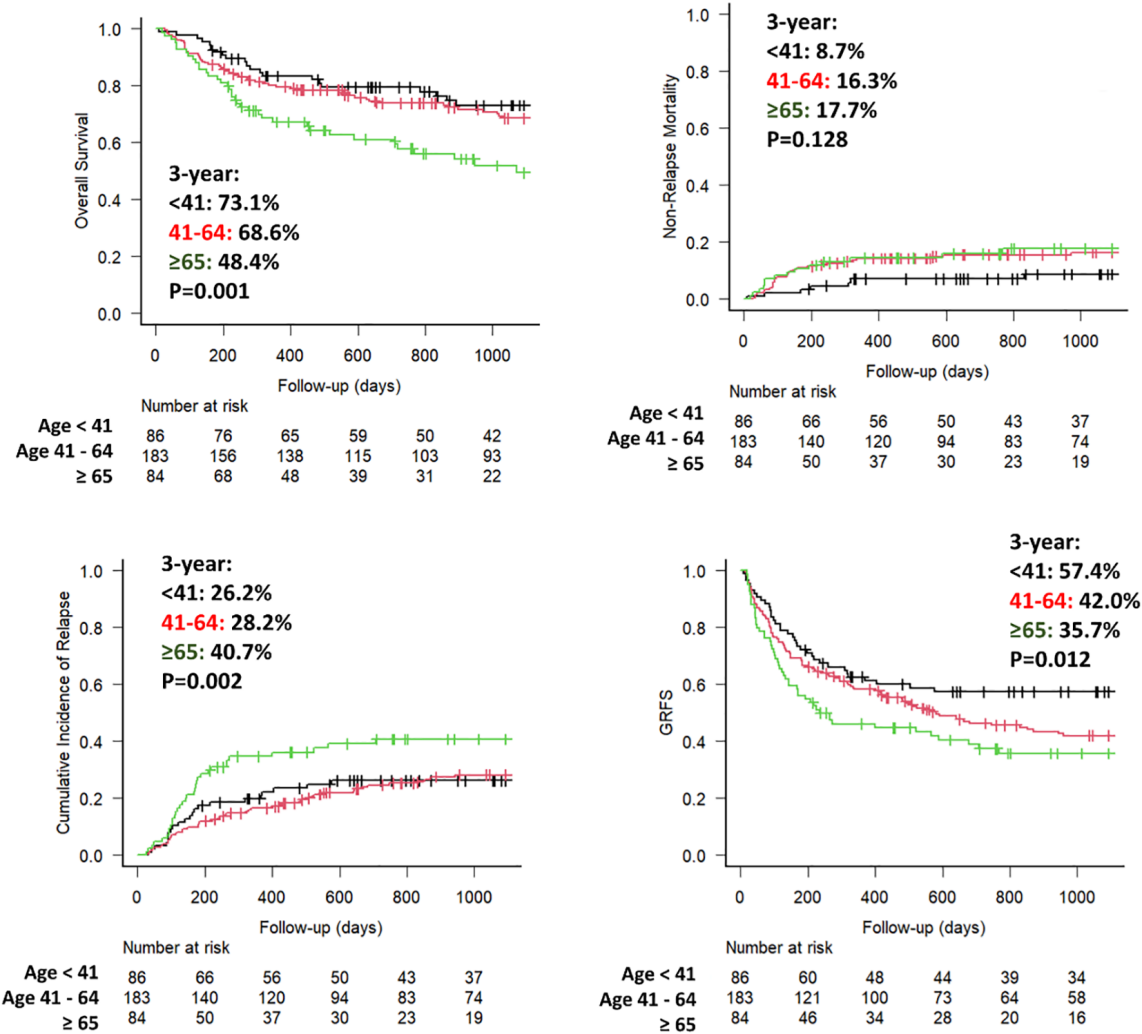
Main outcomes according to age ranges. Overall survival (OS), non-relapse mortality (NRM), cumulative incidence of relapse (CIR), and GVHD-free/relapse-free survival (GRFS) according to age groups (younger ≤40 years, middle-aged 41–64 years, older ≥65 years).

Stratified by age groups, the 2-year OS rates were 57.9% for older, 73.9% for middle-aged, and 79.5% for younger patients. Correspondingly, the 2-year NRM and CIR rates were 16.0% and 40.7% for older, 15.6% and 24.6% for middle-aged, and 7.1% and 26.2% for younger patients (**Table 3**, **Figure 3**). Lastly, GRFS rates were estimated according to age groups showing that at 2 years were respectively, 37.4% for older, 46.3% for middle-aged, and 57.4% for younger patients (P = 0.012).

Lastly, the impact of age groups in outcomes was further investigated using UVA and MVA and summarized in **Table 3**. UVA revealed that, compared with older patients, younger patients had higher probability of OS (HR 0.41, P<0.001), a reduced risk of NRM (HR 0.42, P = 0.057), and a lower probability of disease relapse (HR 0.56, P = 0.046). Subsequently, the results observed in the UVA were confirmed in the MVA controlling for variables found to be significant in the UVA model and those considered relevant for transplant success, confirmed that younger patients maintained a significantly higher OS (HR 0.48, P = 0.024), primarily due to a lower risk of NRM (HR 0.26, P = 0.004) compared with older recipients.

**Table 3:** Risk factors for OS, NRM and CIR.

Univariate analysis	Overall survival		Non-relapsemortality		Cumulative incidence of relapse	
	HR (95% CI)	P value	HR (95% CI)	P value	HR (95% CI)	P value
Age ranges						
≤40y (vs. ≥65 y)	0.41 (0.24-0.70)	0.001	0.42 (0.17-1.02)	0.057	0.56 (0.32-0.98)	0.046
41–64 y (vs. ≥65 y)	0.56 (0.37-0.84)	0.005	0.86 (0.46-1.60)	0.64	0.59 (0.38-0.92)	0.02
Karnofsky performance atatus						
<90% (vs. 90-100%)	2.02 (1.38-2.96)	<0.001	3.01 (1.74-5.23)	<0.001	1.47 (0.95-2.28)	0.78
HCT-CI						
>3 (vs. 0-3)	1.73 (1.17-2.56)	0.006	1.18 (0.63-2.21)	0.6	0.93 (0.57-1.50)	0.08
Disease risk index*						
High/Very High (vs. Low/Int)	2.55 (1.75-3.70)	<0.001	1.55 (0.87-2.77)	0.14	2.02 (1.35-3.01)	<0.001
Conditioning intensity						
RIC (vs. MAC)	1.67 (1.14-2.45)	0.008	1.01 (0.58-1.75)	0.97	1.86 (1.23-2.79)	0.008
Donor type						
10/10 MUD (vs. MSD)	0.83 (0.48-1.44)	0.524	1.94 (0.55-6.72)	0.3	0.50 (0.30-0.83)	0.007
9/10 MMUD (vs. MSD)	1.06 (0.61-1.84)	0.816	4.46 (1.36-14.67)	0.012	0.33 (0.19-0.58)	<0.001
Haploidentical (vs. MSD)	0.75 (0.40-1.41)	0.38	2.91 (0.81-10.39)	0.1	0.33 (0.17-0.61)	0.005

Multivariate analysis	HR (95% CI)	P value	HR (95% CI)	P value	HR (95% CI)	P value
Age ranges						
≤40y (vs. ≥65 y)	0.48 (0.26-0.91)	0.024	0.26 (0.10-0.66)	0.004	0.98 (0.50-1.93)	0.97
41–64 y (vs. ≥65 y)	0.66 (0.42-1.02)	0.064	0.68 (0.34-1.35)	0.28	0.78 (0.48-1.26)	0.32
Karnofsky performance status						
<90% (vs. 90-100%)	2.03 (1.38-2.99)	<0.001	3.38 (1.93-5.91)	<0.001	N/A	–
HCT-CI						
>3 (vs. 0-3)	1.55 (1.04-2.33)	0.031	1.25 (0.65-2.38)	0.49	N/A	–
Disease risk index*						
. High/Very High (vs. Low/Int)	2.55 (1.74-3.73)	<0.001	N/A	–	2.02 (1.36-3.02)	<0.001
Conditioning intensity						
RIC (vs. MAC)	1.20 (0.76-1.89)	0.414	0.65 (=.36-1.18)	0.17	1.75 (1.06-2.90)	0.027
Donor type						
MMUD/Haplo (vs. MSD/MUD)	1.26 (0.87-1.83)	0.213	2.74 (1.47-5.09)	0.001	0.51 (0.34-0.76)	0.001

*Patients with DRI unclassifiable (such as those diagnosed with non-malignant disorders, were classified as low/intermediate risk).

HCT-CI, Hematopoietic Cell Transplant – Comorbidity Index; RIC, Reduced Intensity Conditioning; MAC, Myeloablative Conditioning; MUD, Matched Unrelated Donor; MSD, Matched Sibling Donor; MMUD, Mismatched Unrelated Donor.

In addition, the MVA revealed that impaired performance status (KPS <90%) was associated with both inferior OS (HR 2.03, P<0.001) and higher NRM (HR 3.38, P<0.001). Higher comorbidity burden (HCT-CI >3) correlated with worse OS (HR 1.55, P = 0.031), and patients with high/very-high DRI showed significantly poorer OS (HR 2.55, P<0.001) secondary to an increased risk of relapse (HR 2.02, P<0.001). Lastly, donor type also played a critical role, as the selection of mismatched donors was associated with higher NRM (HR 2.74, P = 0.001).

## Discussion

The present study analyzes the impact of age on allo-HCT outcomes and toxicity profiles in the context of universal PTCy-based GVHD prophylaxis. Our data show that patients older than 65 years had significantly shorter OS, primarily due to increased NRM, with an additional contribution from disease relapse. Nevertheless, our findings support the feasibility of allo-HCT with PTCy in elderly patients, as evidenced by similar engraftment kinetics, comparable early transplant-related complication rates, and similar outcomes when compared with those obtained in middle-age patients undergoing transplantation.

Given the increasing use of PTCy and the growing number of older patients undergoing allo-HCT, evaluating its safety and efficacy in this population is critical. In a previous analysis conducted by our group, we reported a 2-year OS of 45.5% and an NRM 27.1% in the 57 patients ≥65 years who underwent allo-HCT with PTCY at our institution since its implementation (14). In this expanded study that contains information on 84 patients, we observed a 3-year OS of 49.4% in this age group, consistent with recent registry-based analyses from the EBMT and GETH-TC (15–17). For instance, Heidenreich et al. reported a 3-year OS of 34% in patients ≥70 years with MDS or secondary AML, while Maffini et al. described 2-year OS rates of 47–74% in AML patients not in remission. Similarly, Fernández-Luis et al. found a 4-year OS of 43.4% and an NRM of 32% in patients ≥65 years.

Lastly, and aligned with that has been observed in the present investigation, Abedin et al. recently reported a subanalysis of the BMT CTN 1703 trial (NCT03959241) evaluating GVHD prophylaxis in 96 adults aged ≥70 years undergoing RIC allo-HCT (10). Results showed that PTCy-based prophylaxis significantly GRFS (67.1% vs 29.5%), reduced 1-year NRM, and yielded superior 1-year OS (94.3% vs 60.2%) compared with tacrolimus/methotrexate, and supported the use of PTCy in older allo-HCT recipients.

Notably, compared with younger adults, NRM rates were higher in middle-age and older patients, but comparable between these two groups despite the higher comorbidity burden documented in older patients. This result is particularly significant for clinical practice, as high NRM has historically limited the referral of elderly patients to transplant clinics. Importantly, this relatively low NRM in older adults (17.7%) reflects advances in supportive care, careful patient and donor selection, advances in conditioning platforms and the protective effect of PTCy in preventing severe GVHD (3, 18). Nonetheless, infections remained the leading cause of NRM, emphasizing the need for robust infection surveillance and preemptive treatment strategies in this population (19–22).

A key finding was the higher CIR observed in elderly patients in the UVA; however, this effect was not confirmed in the MVA after adjusting for conditioning intensity, suggesting that the predominance of RIC regimens in this subgroup may account for the observed trend. Since RIC may compromise long-term disease control in high-risk hematologic malignancies (23–26), our results emphasize the need to optimize conditioning intensity, donor selection, consider post-transplant maintenance strategies, and tailor immunosuppression tapering to reduce relapse risk while minimizing toxicity in older adults undergoing transplantation.

Interestingly, the incidence of aGVHD did not differ significantly across age groups, supporting the consistent efficacy of PTCy in preventing early GVHD regardless of age. These findings align with prior retrospective studies and prospective trials which also included, among others, older patients undergoing allo-HCT, which demonstrated robust GVHD prevention with PTCy-based regimens (5–9). However, GVHD still contributed to morbidity and also, mortality in some patients, emphasizing the need for continuous refinement of prophylactic strategies and early and effective intervention protocols.

An important observation in our study was the lower incidence of moderate-to-severe cGVHD in patients older than 65 years (3.1%) compared to other groups (younger 4.8% and middle age 12.1%). This aligns with previous studies reporting reduced GVHD severity in older patients, possibly due to age-related immune changes and reduced T-cell reactivity, especially if older adults received grafts from older sibling donors. The use of PTCy may have further contributed to these low cGVHD rates. Nevertheless, while reduced cGVHD is beneficial for quality of life, it may be associated with diminished graft-versus-leukemia effects and a higher relapse risk, as reflected in our CIR findings. Future prospective studies evaluating alternative GVHD prophylaxis or post-transplant maintenance therapies are needed to minimize relapse while maintaining low toxicity.

Regarding infectious complications, we noted a trend toward higher early BSI in middle-aged and older patients, although these differences were not statistically significant. This trend may reflect a higher burden of comorbidities in older patients, potentially increasing susceptibility despite adequate prophylactic strategies. CMV reactivation was frequent but decreased significantly following the introduction of letermovir prophylaxis in 2021. Notably, a higher proportion of patients in the older cohort received letermovir (as were transplanted after 2021), contributing to the lower incidence of CMV reactivation observed in this group. These findings further support the efficacy of CMV-prophylaxis in older patients undergoing allo-HCT with PTCy. Other infectious complications, such as BK virus hemorrhagic cystitis, HHV-6 reactivation, and invasive fungal infections, were not significantly influenced by age, suggesting that standardized prophylaxis protocols and vigilant monitoring reasonably control these risks across age groups. Cardiac toxicity tends to be higher in patients older than 40 years probably due to the higher prevalence of cardiovascular risk factors on those patients, and ICU admissions were also comparable between age groups, supporting the overall safety of allo-HCT with PTCy in elderly patients.

The retrospective design is considered the main limitation of the present study. Moreover, our analysis did not incorporate biological markers of aging such as frailty indices, inflammation markers, or clonal hematopoiesis status, which may more accurately reflect physiologic age and predict transplant outcomes. However, results are considered particularly relevant given the steady increase in the number of older patients undergoing allo-HCT and the expanding use of PTCy-based prophylaxis in this population.

The present study provides valuable real-world evidence supporting the safety and feasibility of PTCy in elderly transplant recipients and informs clinicians on age-related risks and outcomes. Importantly, age alone should not be considered an absolute contraindication to allo-HCT. Supporting this, data from the CIBMTR and EBMT registries demonstrate a progressive rise in transplant recipients older than 65 years over recent decades. This growing trend underscores the need to refine conditioning approaches, optimize GVHD prophylaxis, and integrate personalized maintenance strategies to improve long-term outcomes in this vulnerable but expanding patient group.

In conclusion, our study supports the feasibility and safety of allo-HCT with PTCy-based prophylaxis in older patients, demonstrating acceptable engraftment rates, manageable toxicity, and competitive survival outcomes. Nonetheless, age remains a significant prognostic factor, influencing relapse risk and overall survival. Our findings highlight the need for age-adapted strategies, including careful conditioning selection, optimized GVHD prophylaxis, and robust infection management, to further improve outcomes in this growing patient population.

## Data Availability

The original contributions presented in the study are included in the article/**Supplementary Material**. Further inquiries can be directed to the corresponding author.
