# Supplementary material for: Allo-HCT with post-transplant cyclophosphamide in older adults: similar safety and a viable option compared to younger adults
Source: Front Immunol. 2026 Jan 7;16:1678899. doi: 10.3389/fimmu.2025.1678899 (PMC12819587; doi:10.3389/fimmu.2025.1678899)
Supplement: Supplementary file 1 [file Supplementaryfile1.docx]

**SUPPLEMENTARY MATERIAL**

General criteria for pre-transplant organ function include left ventricular ejection fraction ≥40% without significant pre-existing cardiac disease or uncontrolled arrhythmia; pulmonary function testing demonstrating diffusing capacity of carbon monoxide >40% predicted; normal/stable kidney function; and liver functions tests showing total bilirubin <2.5 times normal with transaminases <3 times the upper limit of normal.

Engraftment after allo-HCT was defined as the presence of an absolute neutrophil count greater than ≥0.5 x 10^9^/L on the first of three consecutive days. Platelet recovery was defined as a sustained platelet count > 20 x 10^9^/L (1st of 3 days) without platelet transfusion for 7 days. Primary graft failure was defined as peripheral blood ANC < 0.5×10^9^/L by day+28 after allo-HSCT in the absence of relapse and secondary graft failure was defined as loss of donor chimerism (<5% donor cells), in whole blood, after initial engraftment and recurrent ANC < 0.5×10^9^/L.
